# Study on mechanism of differential concentration corrosion

**DOI:** 10.1038/s41598-020-75166-7

**Published:** 2020-11-06

**Authors:** Bin Liu, Yingwei Liu

**Affiliations:** 1 Corrosion and Protection Laboratory, Key Laboratory of Superlight Materials and Surface Technology, Ministry of Education, Harbin Engineering University, Harbin, 150001 China; 2 Corrosion and Protection Division, Shenyang National Laboratory for Materials Science, Northeastern University, Shenyang, 110819 China

**Keywords:** Engineering, Materials science

## Abstract

The pipeline easily gets corroded in a seawater environment. The oxygen in the seawater is one of major parameters causing the corrosion. In practice, the corrosion due to the oxygen concentration difference, i.e. differential concentration corrosion (DCC), cannot be avoided. However, a one-dimensional DCC model cannot satisfactorily predict the corrosion because the oxygen distribution near the pipe wall is two-dimensional. In this regard, a two-dimensional DCC model was proposed in this study to numerically investigate the distribution of corrosion potential and current in the ionic conductive layer near the pipe wall as well as the overall corrosion current. The results show that DCC plays a significant role in determining the corrosion potential and current. Without considering DCC, a large corrosion potential and current exist at the location with high oxygen concentration near the pipe wall, whereas the occurrence of the low corrosion potential and current corresponds to the location with low oxygen concentration. However, as DCC is considered, at the location with high corrosion potential, cathodic polarization was produced and the corrosion rate decreases; at the location with low corrosion potential, anodic polarization was produced and the corrosion rate increases. In general, the corrosion potential can be homogenized in terms of DCC.

## Introduction

The differential concentration corrosion (DCC), which is due to the non-uniform distribution of oxygen concentration in an electrolyte^1^, is an important corrosion mechanism. For example, when a structure is immersed in seawater, the corrosion potential at its upper part is higher than that of the lower part because the oxygen concentration near the surface of seawater is higher than that in deep seawater. The potential difference generated between the upper and lower parts of the structure can lead to the transmission of electrons and subsequent corrosion on the structure.

Generally, DCC occurs only when the oxygen concentration difference reaches a certain level. In reality, it is not easy to measure the oxygen concentration throughout the solution. Therefore, DCC was always ignored in the engineering analysis. However, in a complex environment where the distribution of oxygen is highly uneven, the DCC should be considered to help explain the mechanism for some peculiar corrosion phenomena. For example, Matsumura^2^ studied the failure of pipelines in Japanese Mihama Nuclear Power Plant. It was found that the outer elbows of these pipelines became thinner and thinner and were even damaged. It is difficult to explain the result by using traditional flow accelerated corrosion theory (FAC). Traditional theory^3^ indicates that the bend in the pipeline has a large shear stress due to a fast fluid flow rate leading to a thinner boundary layer and a higher concentration of oxygen. Thus, the oxygen concentration gradient is larger, causing a higher mass transfer rate and higher electrochemical reaction rate. Correspondingly, the corrosion rate increases. Based on this traditional theory, the internal side of pipeline bend should be corroded firstly. However, the fact is contrary to the above estimation, the external elbow of the elbow pipe is first reduced and even damaged. The contradiction result only can be explained by the DCC mechanism.

In the past, this kind of research work^4–11^ was carried out under laboratory conditions, and the oxygen concentration in the container was evenly distributed, which could not be applied to engineering. In practice, the distribution of oxygen is more complicated than that in the experimental conditions. For example, the fluid in the seawater pipeline is dissolved with a certain amount of oxygen, significant turbulence tends to cause a complex distribution of oxygen when the fluid flows in the pipeline. The oxygen distribution is very non-uniform even if the shape of a pipeline is not complex. The oxygen distribution can be non-uniform in both axial and circumferential directions. Laboratory conditions cannot meet the needs of engineering practice. In this regard, numerical simulation was applied to study the DCC under complex situations. For example, Lu et al.^12^ proposed a model to predict the reducing-pipe flow accelerated corrosion. The concept of DCC was introduced into the traditional FAC model based on the difference in oxygen concentration at each end of a reducing pipe. The corrosion rate of the reducing pipe section was then calculated. Zhu et al.^13^ predicted the corrosion rate of the loop pipeline elbow in a nuclear power plant, using a DCC model based on oxygen concentration difference between the inner bend and external elbow. There is a very thin ionic conductive layer between the inner bend and external elbow according to the model, forming an electronic conduction loop within the pipeline. An analytical method proposed by Song^14^ was adopted to calculate the corrosion rate at several points along the conductive path between inner bend and the external elbow. The result indicates that the corrosion current considering DCC is higher than that without DCC by one order of magnitude. Therefore, the elbow can be corroded and damaged earlier. The verified result obtained by Masumura^2^ indicates that macro-battery corrosion caused by concentration difference must be considered when the distribution of oxygen is not uniform to a certain extent.

Previous numerical studies on the corrosion of pipeline have almost exclusively used one-dimensional model assuming a uniform distribution of oxygen. However, such an approximate analysis is not sufficient. In practice, the distribution of oxygen is not uniform. Therefore, considering uneven distribution of the oxygen along the axial and circumferential directions in the pipeline, in this study, a two-dimensional DCC model was proposed to numerically investigate the distribution of corrosion potential and current in the ionic conductive layer near the pipe wall as well as the overall corrosion current. .

## Differential concentration corrosion mechanism

**Figure 1:**
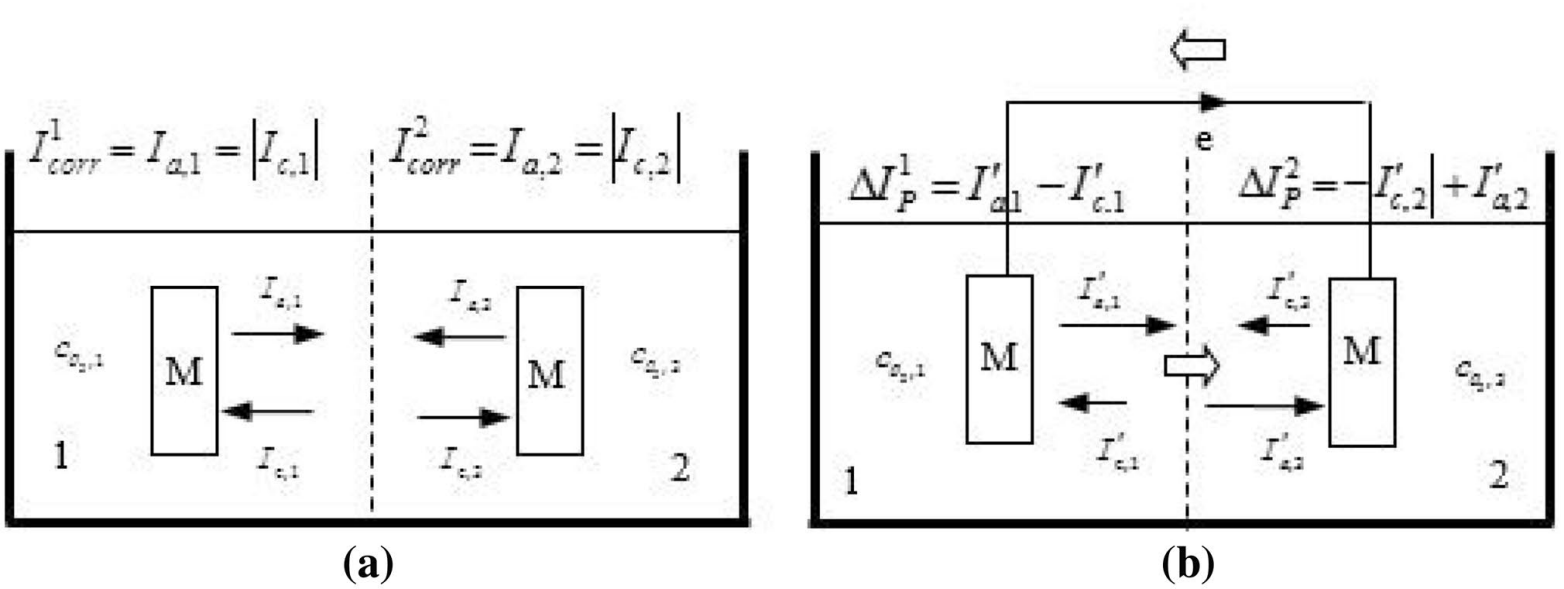
A galvanic cell for corrosion (**a**) two independent electrochemical reactions, and (**b**) coupled electrochemical reactions.

The corrosion process can be explained using the following experiment. As illustrated in Fig. 1a, semi-permeable membrane is used for dividing a galvanic cell containing electrolytes into two parts. Two iron sheets with the same properties are submerged into both parts. Different amounts of oxygen are injected into both sides through a particular device to make different oxygen concentrations, i.e. $c_{{o_{2,2} }} > c_{{o_{2,1} }}$. The following electrochemical reactions can occur at both parts:

1 $${\text{Fe}} \to 2{\text{Fe}}^{2 + } + 4{\text{e}}$$

2 $${\text{O}}_{2} + 2{\text{H}}_{2} {\text{O}} + 4{\text{e}} \to 4{\text{OH}}^{ - 1}$$

The equilibrium potential, exchange current density and Tafel slope at both parts, i.e. $E_{e,a} ,I_{0,a} ,\beta_{a}$ and $E_{e,c} ,I_{0,c} ,\beta_{c}$, are listed in Table 1, respectively. The coupling of reactions (1) and (2) leads to a mixed potential, ${E}_{corr}$, and corrosion current, ${I}_{corr}$. In general, ${E}_{corr}$ is far different from the equilibrium potential ${E}_{e,a},{E}_{e,c}$, so the contraries of the two reactions can be ignored. In addition, the control step of the whole reaction is determined by the discharge process if the concentration of oxygen appropriately increases. Therefore, the above coupling reaction can be described by the simplified Butler–Volmer formula:

3 $$I_{a} = I_{0,a} \exp \left( {\frac{{E_{corr} - E_{e,a} }}{{\beta_{a} }}} \right)$$

4 $$I_{c} = - I_{0,c} \exp \left( {\frac{{E_{e,c} - E_{corr} }}{{\beta_{c} }}} \right)$$

**Table 1:** Electrochemical parameters.

Parameters	Iron	Oxgyen
Equilibrium potential (V)	− 0.76	0.189
Tafel slope (V/m)	0.41	0.18
Exchange current degree (A/m^2^)	7.7 × 10^–7^	7.1 × 10^–5^
Conductivity of seawater (S)	4	

Consequently, ${E}_{corr}$ and ${I}_{corr}$ can be obtained by combining Eqs. (3) and (4) due to $\left|{I}_{c}\right|={I}_{a}={I}_{corr}$:

5 $$E_{corr} = \frac{1}{{\beta_{a} + \beta_{c} }}\left[ {\left( {\beta_{a} E_{e,c} + \beta_{c} E_{e,a} } \right) - \beta_{a} \beta_{c} \ln \left( {\frac{{I_{0,a} }}{{I_{0,c} }}} \right)} \right]$$

6 $$I_{corr} = I_{0,a}^{{\frac{{\beta_{a} }}{{\beta_{a} + \beta_{c} }}}} I_{0,c}^{{\frac{{\beta_{c} }}{{\beta_{a} + \beta_{c} }}}} \exp \left( {\frac{{E_{e,c} - E_{e,a} }}{{\beta_{a} + \beta_{c} }}} \right)$$

The electrochemical reactions on both parts of the galvanic cell can be described using Eqs. (5) and (6) in Fig. 1a. The equilibrium potential, ${E}_{e,c}$, of electrode reaction (2) and the exchange current density, ${I}_{0,c}$, vary with different oxygen concentrations on both parts. Based on Nernest equation^15^, the equilibrium potential and exchange current density can be calculated as follows:

7 $$E_{e,c}^{1} = E_{e,c}^{{}} + \frac{RT}{{nF}}\ln \left( {\frac{{c_{{o_{2,1} }} }}{{c_{{o_{2} ,0}} }}} \right)$$

8 $$I_{0,c}^{1} = I_{0,c}^{{}} \left( {\frac{{c_{{o_{2,1} }} }}{{c_{{o_{2} ,0}} }}} \right)$$

9 $$E_{e,c}^{2} = E_{e,c}^{{}} + \frac{RT}{{nF}}\ln \left( {\frac{{c_{{o_{2,2} }} }}{{c_{{o_{2} ,0}} }}} \right)$$

10 $$I_{0,c}^{2} = I_{0,c}^{{}} \left( {\frac{{c_{{o_{2,2} }} }}{{c_{{o_{2} ,0}} }}} \right)$$

where ${c}_{{o}_{2},0}$ is the oxygen concentration at inlet, corresponding to the equilibrium potential ${E}_{e,c}$ ,and its value is listed in Table 2. Equations (7)–(10) can been substituted into Eqs. (5) and (6), and then the corrosion potentials ${E}_{corr}^{1}$ and ${E}_{corr}^{2}$ as well as the corrosion current ${I}_{corr}^{1}$ and ${I}_{corr}^{2}$ on both parts can be obtained, respectively. This is the corrosion occurred on both parts when sampe 1 and 2 are not connected.

**Table 2:** Boundary conditions used in simulation.

Velocity on inlet (m/s)	Outlet pressure (Pa)	$c_{{o_{2} }} ,0$ (m^3^ m^−3^)
0.3	1.013 × 10^5^	3.854 × 10^–3^

When parts 1 and 2 are connected using wires as illustrated in Fig. 1b, in the condition of ${E}_{corr}^{2}>{E}_{corr}^{1}$, the electrons flow through a wire from part 1–2 due to potential difference, and the ion directional movement also occurs in the solution. Thus, the external current is generated. The corrosion potential. leads to polarization, i.e. ${E}_{corr}^{2}$ decreases and ${E}_{corr}^{1}$ increases,which help produce different polarization current $\Delta {I}_{P}^{1}$ and $\Delta {I}_{P}^{2}$. the change of corrosion current of parts 1 and 2 can be described as ${{I}^{^{\prime}}}_{corr}^{1}={I}_{corr}^{1}+\Delta {I}_{P}^{1}$ and ${{I}^{^{\prime}}}_{corr}^{2}={I}_{corr}^{2}+\Delta {I}_{P}^{2}$.

## Two-dimensional DCC model

### Calculation of oxygen distribution

**Figure 2:**
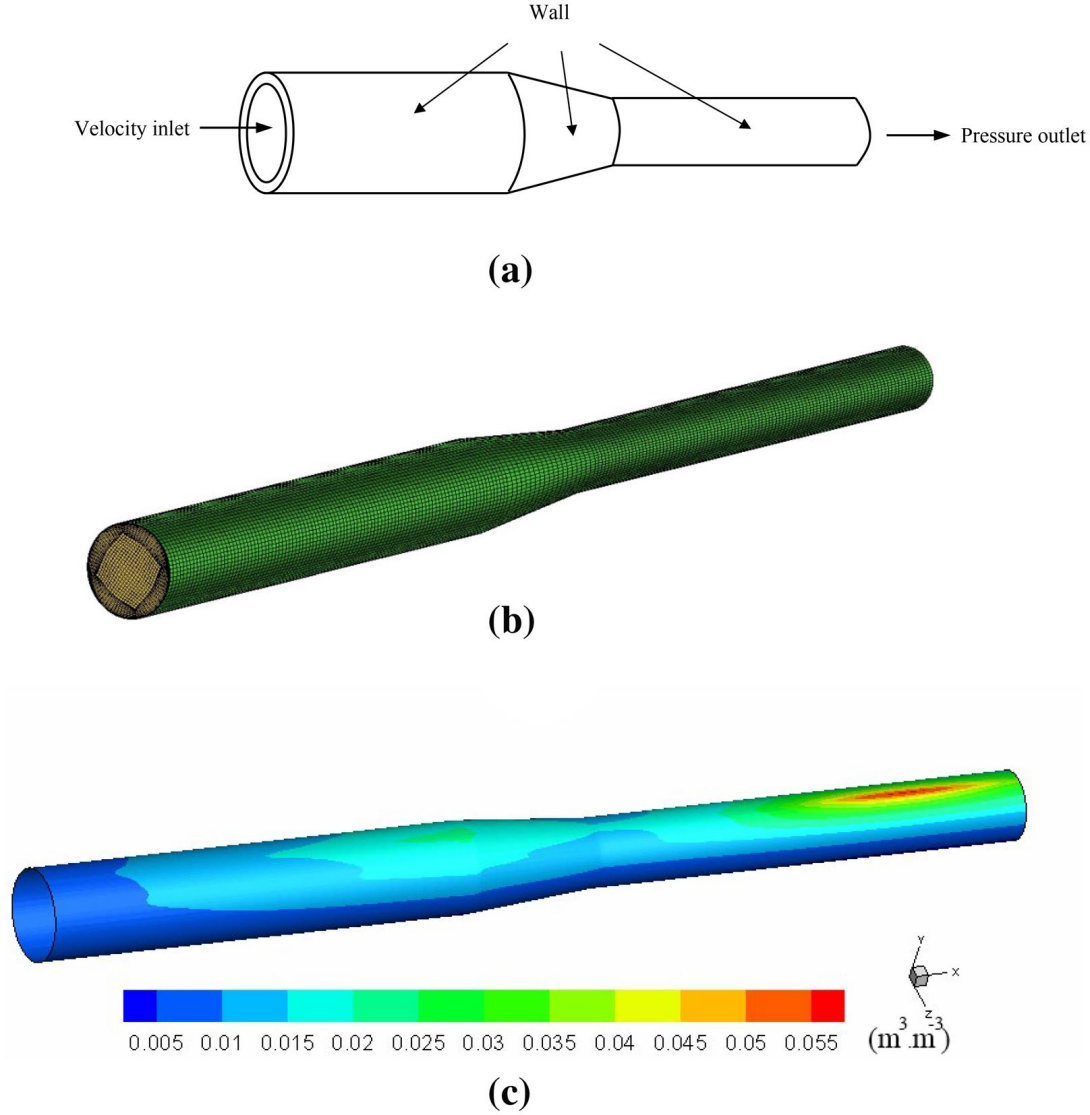
Simulation of seawater flow in the pipeline: (**a**) boundary conditions, (**b**) computational mesh and (**c**) calculated oxygen distribution near the wall.

The generative mechanism of the DCC was introduced using the aforementioned experimental devices. But in practice, the corrosion is more complex. The reduced pipeline,depicted in Fig. 2a, is used to help explain the corrosion mechanism in complex conditions.

The oxgen distribution near the wall was simulated in ANSYS FLUENT 16.0 before carrying out DCC modelling,a multiphase flow model of mixure was applied. As shown in Fig. 2a, the inlet velocity and oxygen content, ${c}_{{o}_{2},0}$, and outlet pressure were set. The values are given in Table 2. No sliding wall condition was applied. The turbulent flow in the pipeline was simulated by κ-ε model. Wall function method was used with the dimensionless distance, $y^{ + } = 50$, which refers to the distance between the center point of the first layer element and the wall surface. Figure 2b,c show the computational mesh and oxygen distribution near the wall, respectively.

**Figure 3:**
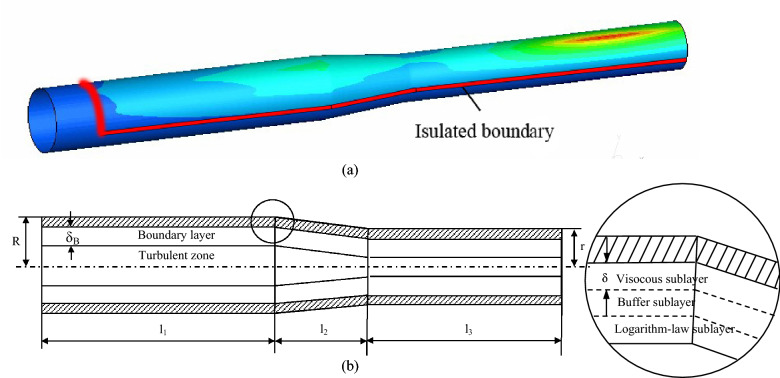
Computational domain used for DCC modeling: (**a**) a division between upper (selected for DDC simulation) and lower parts of pipeline and (**b**) schematic of boundary layer.

Due to the low density of oxygen, under the action of gravity, most of the oxygen is concentrated in the upper part of the pipeline (Fig. 2c). The results show that the oxygen concentration in the upper part is 3 to 10 times higher than that in the lower part. Therefore, to simplify the simulation condition, the lower part of the pipeline was neglected. Only upper part of the pipeline was used in the subsequent simulation while the boundary between the lower and upper part is regarded as the insulation boundary, as illustrated in Fig. 3a. The corresponding near wall boundary layer is given in Fig. 3b.

### Numerical model of two-dimensional DCC

**Figure 4:**
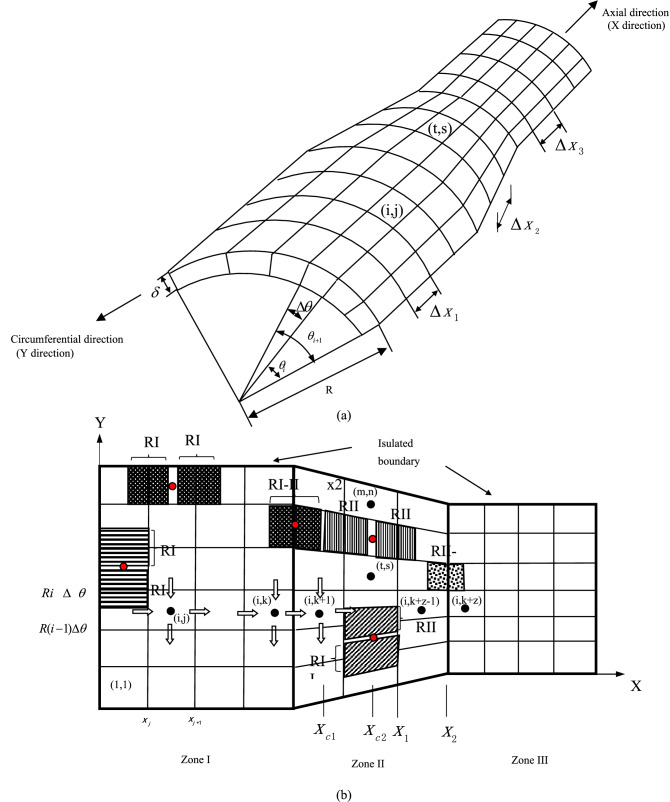
Schematic of discretization of viscous sublayer: (**a**) element division and (**b**) expanded view of viscous sublayer.

Viscous sublayer was used to understand DCC in the pipeline. The schematic of discretization of viscous sublayer is shown in Fig. 4a. Three zones (Zone I, II and III) can be identified.

**Figure 5:**
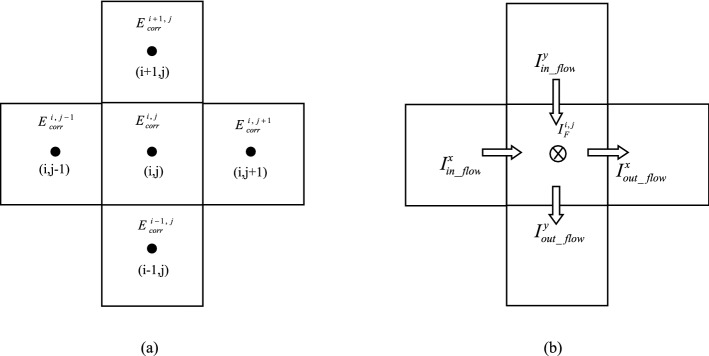
Potential and current flow between elements: (**a**) corrosion potential of central element and its neighbors, and (**b**) current flow between elements.

**Figure 6:**
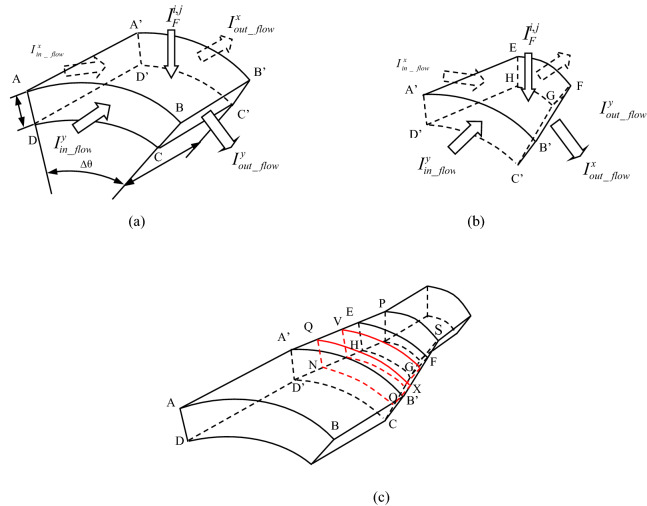
Basic elements in different zones: (**a**) element in Zone I and III, (**b**) element in Zone II, and (**c**) connection of different regional elements.

As shown in Fig. 5, a representative element (i, j) and its neighboring elements are taken to show potential and current flow between elements. If DCC is not considered, there only will be a natural corrosion potential $E_{corr}^{i,j}$ and a natural corrosion current $I_{corr}^{i,j}$ in each element, where (i, j) stands for arbitrary element number. But in fact, all elements are interconnected with one another, which inevitably generate current. Thus, the element corrosion potential ($E_{corr}^{i,j}$) can be polarized. The polarization potential is expressed as ${E}^{i,j}-{E}_{corr}^{i,j}$ and the polarization current is ${I}_{F}^{i,j}$ (Fig. 6a,b). *E*^*i*,*j*^ is the ultimate corrosion potential of element (i, j) after polarization.

As the elements are connected to each other, the natural corrosion potential difference between them can drive a current, as illustrated in Figs. 4b, 5b, 6a and b. Taking element (i, j) as an example also, as DCC is not considered, the absolute values of anodic reaction current and cathodic current between the wall and element in the solution are equal, and no net current is generated. However, as DCC is considered, the current flow between elements leads to a polarization of the corrosion potential. The anode and cathode currents are no longer equal so that the net current, i.e. external current or polarization current, can be generated. At the same time, other elements around the element (i, j) will also have current flowing in or out, as illustrated in Figs. 4b, 5b, 6a and b. If all elements are considered as a circuit, the element (i, j) can be treated as a node in the circuit. When the current reaches a steady state, according to Kirchhoff's Second Law^16^, a net flow of current through the element is zero.

#### Derivation of discrete equations used in zone I and III

According to the balance of current at a steady state, an equation of the corrosion potential of the polarized element can be obtained. As an example, element (i, j) is surrounded by four neighboring elements. Since each element represents an electrolyte, according to the definition of corrosion potential, ${E}^{i,j}$ is the potential difference between the wall and the solution and $-{E}^{i,j}$ represents the potential difference between solution and wall. Therefore, the current flowing from element (i, j − 1) to (i, j) should be

11 $$I_{in\_flow}^{x} = \frac{{\left( { - E_{{}}^{i,j - 1} } \right) - \left( { - E_{{}}^{i,j} } \right)}}{{R_{x1}^{{\text{I}}} }}$$

Similarly, the current from element (i, j) to element (i, j + 1) is

12 $$I_{out\_flow}^{x} = \frac{{\left( { - E_{{}}^{i,j} } \right) - \left( { - E_{{}}^{i,j + 1} } \right)}}{{R_{x2}^{{\text{I}}} }}$$

Along the Y direction, the current flowing from element (i + 1, j) to element (i, j) is

13 $$I_{in\_flow}^{y} = \frac{{\left( { - E_{{}}^{i + 1,j} } \right) - \left( { - E_{{}}^{i,j} } \right)}}{{R_{y1}^{{\text{I}}} }}$$

The current from element (i, j) to element (i − 1, j) is

14 $$I_{out\_flow}^{y} = \frac{{\left( { - E_{{}}^{i,j} } \right) - \left( { - E_{{}}^{i - 1,j} } \right)}}{{R_{y2}^{{\text{I}}} }}$$

The Faraday current due to polarization is

15 $$I_{F}^{i,j} = \frac{{E_{{}}^{i,j} - E_{corr}^{i,j} }}{{R_{P}^{i,j} }}S_{{AA^{\prime}B^{\prime}B}}$$

where ${R}_{P}^{i,j}$ is the polarization resistance of element (i, j), Ω m^2^, more details please see “Discrete equations of elements at the boundary between zones I and II” section. ${S}_{A{A}^{^{\prime}}{B}^{^{\prime}}B}\approx R\Delta \theta \Delta {x}_{1}$, as illustrated in Fig. 6a. ${R}_{x1}^{{\rm I}}$,${R}_{x2}^{{\rm I}}$,${R}_{y2}^{{\rm I}}$,${R}_{y1}^{{\rm I}}$ is the resistance between elements of the solution in X and Y direction , more details also can be found in “Discrete equations of elements at the boundary between zones I and II” section.

The polarization current is assumed to flow from wall to element. According to Kirchhoff's Second Law, for each element (i, j), there exist

16 $$\sum I_{in} = \sum I_{out}$$

Substitute (11)–(15) into Eqs. (16), (17):

17 $$- \frac{{E_{{}}^{i - 1,j} }}{{R_{{{\text{y2}}}}^{{\text{I}}} \, }} - \frac{{E_{{}}^{i,j - 1} }}{{R_{{{\text{x1}}}}^{{\text{I}}} }} + \left( {\frac{1}{{R_{{{\text{x1}}}}^{{\text{I}}} }} + \frac{1}{{R_{{{\text{x2}}}}^{{\text{I}}} }} + \frac{1}{{R_{{{\text{y1}}}}^{{\text{I}}} }} + \frac{1}{{R_{{{\text{y2}}}}^{{\text{I}}} }} + \frac{{S_{{AA^{\prime}B^{\prime}B}} }}{{R_{P}^{i,j} }}} \right)E_{{}}^{i,j} - \frac{{E_{{}}^{i,j + 1} }}{{R_{{{\text{x2}}}}^{{\text{I}}} }} - \frac{{E_{{}}^{i + 1,j} }}{{R_{{{\text{y1}}}}^{{\text{I}}} }} = \frac{{S_{{AA^{\prime}B^{\prime}B}} }}{{R_{P}^{i,j} }}E_{corr}^{i,j}$$

The corresponding discrete equations can be obtained by similar treatment of other elements. However, due to the different locations of the elements, the form of the equation can be different. Taking element (1, 1) in Fig. 4d as an example, because the left and the bottom of the element are insulated boundaries, ${I}_{out\_flow}^{y}$ and ${I}_{in\_flow}^{x}$ are zero, respectively.When Kirchhoff’s Second Law is applied to the element, the discrete equation becomes

18 $$\left( {\frac{1}{{R_{{{\text{x2}}}}^{{\text{I}}} }} + \frac{1}{{R_{{{\text{y1}}}}^{{\text{I}}} }} + \frac{{S_{{AA^{\prime}B^{\prime}B}} }}{{R_{P}^{1,1} }}} \right)E_{{}}^{1,1} - \frac{{E_{{}}^{1,2} }}{{R_{{{\text{x2}}}}^{{\text{I}}} }} - \frac{{E_{{}}^{2,1} }}{{R_{{{\text{y1}}}}^{{\text{I}}} }} = \frac{{S_{{AA^{\prime}B^{\prime}B}} }}{{R_{P}^{1,1} }}E_{corr}^{1,1}$$

The elements at other boundaries can be treated similarly.

#### Derivation of discrete equations used in zone II

The Kirchhoff Second Law is applied to element (t, s) in Zone II as illustrated in Fig. 4b to obtain discrete equations similar to Eq. (17).

19 $$- \frac{{E_{{}}^{t - 1,s} }}{{R_{{{\text{y2}}}}^{{{\text{II}}}} }} - \frac{{E_{{}}^{t,s - 1} }}{{R_{{{\text{x1}}}}^{{{\text{II}}}} }} + \left( {\frac{1}{{R_{{{\text{x1}}}}^{{{\text{II}}}} }} + \frac{1}{{R_{{{\text{x2}}}}^{{{\text{II}}}} }} + \frac{1}{{R_{{{\text{y1}}}}^{{{\text{II}}}} }} + \frac{1}{{R_{{{\text{y2}}}}^{{{\text{II}}}} }} + \frac{{S_{{A^{\prime}EFB^{\prime}}} }}{{R_{P}^{t,s} }}} \right)E_{{}}^{t,s} - \frac{{E_{{}}^{t,s + 1} }}{{R_{{{\text{x2}}}}^{{{\text{II}}}} }} - \frac{{E_{{}}^{t + 1,s} }}{{R_{{{\text{y2}}}}^{{{\text{II}}}} }} = \frac{{S_{{A^{\prime}EFB^{\prime}}} }}{{R_{P}^{t,s} }}E_{corr}^{t,s}$$

where $R_{{{\text{x1}}}}^{{{\text{II}}}}$ $R_{{{\text{y1}}}}^{{{\text{II}}}}$ $R_{{{\text{x2}}}}^{{{\text{II}}}}$ $R_{{{\text{y2}}}}^{{{\text{II}}}}$ are the resistances of element of Zone II in X and Y direcion. The detailed expression of them will be found in “Discrete equations of elements at the boundary between zones I and II” section.

It is easy to determine $S_{{A^{\prime}EFB^{\prime}}}$ according to the geometric relationship in Fig. 6a:

$$S_{{A^{\prime}EFB^{\prime}}} = \frac{{[R_{x} \Delta \theta + (R_{x} + dR)\Delta \theta ]\Delta x_{2} }}{2} \approx R_{x} \Delta \theta \Delta x_{2}$$

where $R_{x}$ is the radius at coordinate x of reducing pipe section, its expression is:

20 $$R_{x} = k\left( {x - l_{1} } \right) + R$$

and *k* is $\frac{r-R}{{l}_{2}}$. Other definitions for r, R, l_1_, l_2_ are shown in Fig. 3b.

Equation (19) is a standard form. When the element is located at the boundary, the standard form should be changed. For example, for element (m, n) shown in Fig. 4b, because an insulated boundary is above this element, ${I}_{in\_flow}^{y}$ is zero.As Kirchhoff's Second Law is applied to this element, the equation becomes

21 $$- \frac{{E_{{}}^{m - 1,n} }}{{R_{{{\text{y2}}}}^{{{\text{II}}}} }} - \frac{{E_{{}}^{m,n - 1} }}{{R_{{{\text{x1}}}}^{{{\text{II}}}} }} + \left( {\frac{1}{{R_{{{\text{x1}}}}^{{{\text{II}}}} }} + \frac{1}{{R_{{{\text{x2}}}}^{{{\text{II}}}} }} + \frac{1}{{R_{{{\text{y2}}}}^{{{\text{II}}}} }} + \frac{{S_{{A^{\prime}EFB^{\prime}}} }}{{R_{P}^{m,n} }}} \right)E_{{}}^{m,n} - \frac{{E_{{}}^{m,n + 1} }}{{R_{{{\text{x2}}}}^{{{\text{II}}}} }} = \frac{{S_{{AA^{\prime}B^{\prime}B}} }}{{R_{P}^{m,n} }}E_{corr}^{m,n}$$

The elements at other boundaries in Zone II can be treated similarly.

#### Discrete equations of elements at the boundary between zones I and II

As the elements (i, k) and (i, k + 1) are at the junction of a straight pipe and a variable-diameter pipe, as illustrated in Figs. 4b and 6c,the calculation of resistance is different from that in Zones I or II . Application of Kirchhoff's Second Law to element (i, k) can

22 $$- \frac{{E_{{}}^{i - 1,k} }}{{R_{{{\text{y2}}}}^{{\text{I}}} }} - \frac{{E_{{}}^{i,k - 1} }}{{R_{{{\text{x1}}}}^{{\text{I}}} }} + \left( {\frac{1}{{R_{{{\text{x1}}}}^{{\text{I}}} }} + \frac{1}{{R_{{{\text{x2}}}}^{{\text{I}{-}\text{II}}} }} + \frac{1}{{R_{{{\text{y1}}}}^{{\text{I}}} }} + \frac{1}{{R_{{{\text{y2}}}}^{{\text{I}}} }} + \frac{{S_{{AA^{\prime}B^{\prime}B}} }}{{R_{P}^{i,k} }}} \right)E_{{}}^{i,k} - \frac{{E_{{}}^{i,k + 1} }}{{R_{{{\text{x2}}}}^{{\text{I}{-}\text{II}}} }} - \frac{{E_{{}}^{i + 1,k} }}{{R_{{{\text{y1}}}}^{{\text{I}}} }} = \frac{{S_{{AA^{\prime}B^{\prime}B}} }}{{R_{P}^{i,k} }}E_{corr}^{i,k}$$

For element (i, k + 1), the equation becomes:

23 $$- \frac{{E_{{}}^{i - 1,k + 1} }}{{R_{{{\text{y2}}}}^{{{\text{II}}}} }} - \frac{{E_{{}}^{i,k} }}{{R_{{{\text{x2}}}}^{{\text{I} {-} \text{II}}} }} + \left( {\frac{1}{{R_{{{\text{x2}}}}^{{{\text{II}}}} }} + \frac{1}{{R_{{{\text{x2}}}}^{{\text{I} {-} \text{II}}} }} + \frac{1}{{R_{{{\text{y1}}}}^{{{\text{II}}}} }} + \frac{1}{{R_{{{\text{y2}}}}^{{{\text{II}}}} }} + \frac{{S_{{AA^{\prime}B^{\prime}B}} }}{{R_{P}^{i,k + 1} }}} \right)E_{{}}^{i,k + 1} - \frac{{E_{{}}^{i,k + 2} }}{{R_{{{\text{x2}}}}^{{{\text{II}}}} }} - \frac{{E_{{}}^{i + 1,k + 1} }}{{R_{{{\text{y1}}}}^{{{\text{II}}}} }} = \frac{{S_{{A^{\prime}EFB^{\prime}}} }}{{R_{P}^{i,k + 1} }}E_{corr}^{i,k + 1}$$

where ${R}_{x12}$ is the solution resistance between element (i, k) and element (i, k + 1), ${R}_{x1}$, ${R}_{y1}$, ${R}_{x2}$, and ${R}_{y2}$ can be expressed thoroughly in “Discrete equations of elements at the boundary between zones I and II” section.

### Calculation of resistances

#### Calculation of element resistance in zones I and III

Because the shapes and sizes of the element in three zones are different, the calculation methods of resistance are different. For Zone I, the X-direction resistance of the element is

24 $$R_{x1}^{{\text{I}}} = R_{x2}^{{\text{I}}} = \rho_{s} \frac{{\Delta x_{1} }}{{S_{ABCD} }}$$

The Y-direction resistance is

25 $$R_{y1}^{{\text{I}}} = R_{y2}^{{\text{I}}} = \rho_{s} \frac{R\Delta \theta }{{S_{{BB^{\prime}C^{\prime}C}} }}$$

where

$$\begin{aligned} S_{ABCD} & = \pi \left[ {R^{2} - \left( {R - \delta } \right)^{2} } \right]\frac{\Delta \theta }{{2\pi }} \approx R\delta \Delta \theta \\ S_{{BB^{\prime}C^{\prime}C}} & = \delta \Delta x_{1} \\ \end{aligned}$$

The calculation of resistance of the element in zone III is similar to that in Zone I, with the replacement of R, $\Delta {x}_{1}$ in Eqs. (26) and (27) with r and $\Delta {x}_{3}$.

#### Calculation of element resistance in zone II

Because the width of the element in this zone decreases with increasing values of x, the calculation of resistance is different from that in Zones I and III. As illustrated in Fig. 6c, a typical element is highlighted with the red lines. The total resistance should be the integral of differential resistances between neighbouring elements,

26 $$dR_{x}^{{{\text{II}}}} = \rho_{s} \frac{dx}{{S_{QXON} }}$$

where $${s}_{QXON}=\frac{[{R}_{x}d\theta +({R}_{x}-\delta )d\theta ]\delta }{2}$$

The integral of Eq. (28) gives

27 $$R_{x}^{{{\text{II}}}} = \int\limits_{{x_{c1} }}^{{x_{c2} }} {\frac{{2\rho_{s} dx}}{{\{ 2[k(x - l_{1} ) + R] - \delta \} \Delta \theta \delta }}} = \frac{{2\rho_{s} }}{\Delta \theta k\delta }\ln \frac{{\{ 2[k(x_{c2} - l_{1} ) + R] - \delta \} }}{{\{ 2[k(x_{c1} - l_{1} ) + R] - \delta \} }}$$

where ${x}_{{c}_{1}}$ and ${x}_{{c}_{2}}$ is the center coordinates for two adjacent elements.

In a similar way, the Y-direction resistance can be calculated by an integral method. The resistance is

28 $$dR_{y}^{{{\text{II}}}} = \rho_{s} \frac{R(x)\Delta \theta }{{S_{{B^{\prime}FGC^{\prime}}} }}$$

where $${s}_{{B}^{\mathrm{^{\prime}}}FG{C}^{\mathrm{^{\prime}}}}=\delta dx$$.

The integral of Eq. (30) gives

29 $$R_{y}^{{{\text{II}}}} = = \frac{{k\rho_{s} \Delta \theta }}{\delta }\left[ {\ln \frac{{k\left( {x_{2} - l_{1} } \right) + R}}{{k\left( {x_{1} - l_{1} } \right) + R}}} \right]^{ - 1}$$

where ${x}_{1},{x}_{2}$ are the element start and end coordinates in X-direction.

#### Calculation of element resistance at the boundary between zones I and II

For the elements at the junction of a straight pipe and a variable-diameter pipe, such as elements (i, k) and (i, k + 1), the calculation of resistance between two elements is

30 $$R_{x2}^{{\text{I}{-}\text{II}}} = \frac{1}{2}R_{x2}^{{\text{I}}} + \frac{1}{2}R_{x1}^{{{\text{II}}}}$$

The similar calculation can be done to obtain element resistance at the junction of zones II and III.

#### Calculation of plorization resitance

**T**he polarization resistance between the pipe wall and the solution can be calculated according to the following formula:

31 $$R_{p}^{i,j} = \frac{B}{{I_{i,j}^{corr} }}$$

32 $$B = \frac{{\beta_{a} \beta_{c} }}{{\beta_{a} + \beta_{c} }}$$

where $\beta_{a} ,\beta_{c}$ are the Tafel slope of iron and oxgen respectivly listed in Table 1.

## Results and discussions

Figure 3c illustrates the oxygen concentration distribution near the pipeline wall. It can be seen that in the middle part of the upper wall, the concentration of oxygen is higher than that on both sides. In contrast, along the axial direction, near the outlet of the pipeline, the concentration of oxygen is the highest.

**Figure 7:**
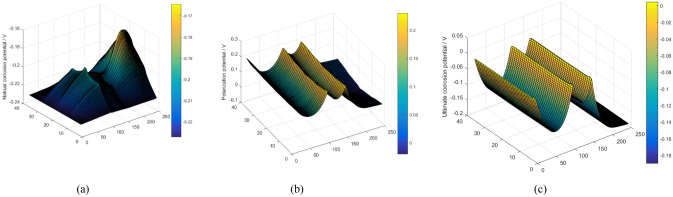
The potential distribution of elements; (**a**) natural corrosion potential distribution without DCC, (**b**) polarization potential distribution considering DCC and(c) ultimate corrosion potential distribution.

**Figure 8:**
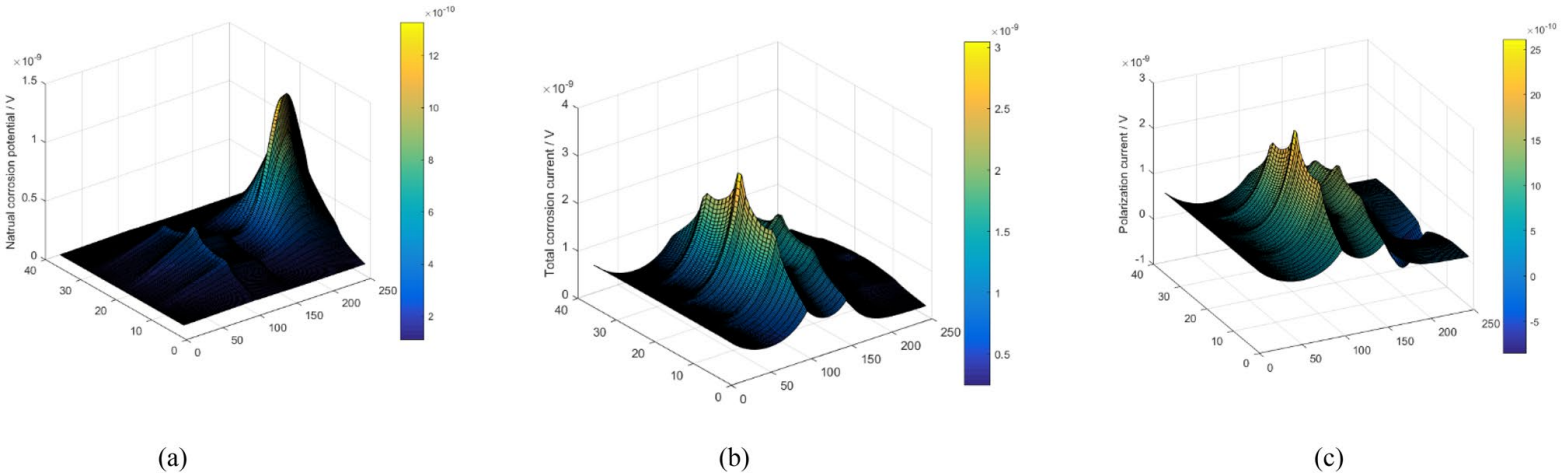
The current distribution of elements; (**a**) natural corrosion current distribution without considering DCC; (**b**) polarization current distribution considering DCC, and (**c**) total corrosion current distribution.

The distribution of oxygen determines the corrosion of the pipeline wall. Figures 7a and 8a show the distribution of natural corrosion potential and current without considering DCC. The distribution of corrosion potential and current is closely in relation to the distribution of oxygen. The high natural corrosion potential and current occurs at location with high oxygen concentration, and vice versa.

However, because the elements are actually connected, the difference in the natural corrosion potentials of the elements will inevitablely lead to a current flow between the elements. Natural corrosion potential is bound to polarize. Moreover, the absolute values of anodic and cathodic reaction current are no longer equal, causing polarization current. Figures 7b and 8b show the distribution of polarization potential and current, respectively. The final corrosion potential and current after polarization are illustrated in Figs. 7c and 8c, respectively.

**Figure 9:**
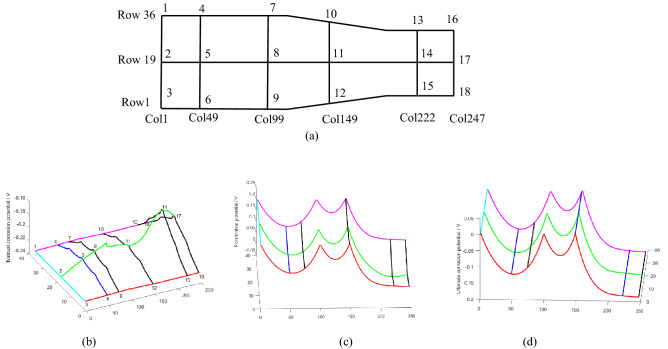
The potential distribution of selected elements: (**a**) selection of elements for study, (**b**) natural corrosion potential distribution without considering DCC, and (**c**) polarization potential distribution considering DCC, and (**d**) ultimate corrosion potential distribution.

**Figure 10:**
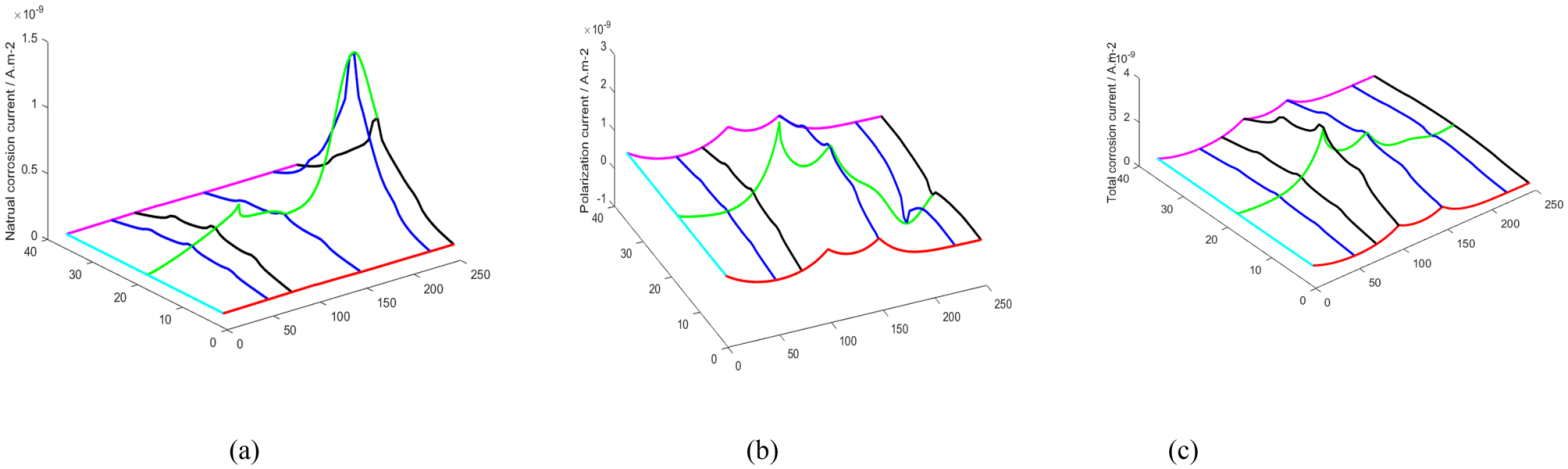
The current distribution of selected elements; (**a**) natural corrosion current distribution without considering DCC, (**b**) polarization current distribution considering DCC, and (**c**) total corrosion current distribution.

The mechanism of concentration corrosion can be described in more details. Figures 9 and 10 illustrate the distribution of the above physical quantities, such as corrosion potential, current et al. at the selected representative rows and columns. Figure 9c shows the polarized potential of each column, indicating the different polarization extents. For example, in the first column, the degree of polarization is the highest and the polarization is anodic. Its means that the corrosion potential increases. Because the natural potential of the element is the lowest, the current mainly flows into the element, resulting in anodic polarization. Similarly, anodic polarization occurs in columns 48 and 99. In comparison to column 1, the higher natural corrosion potential in these two columns causes a lower degree of polarization.

**Figure 11:**
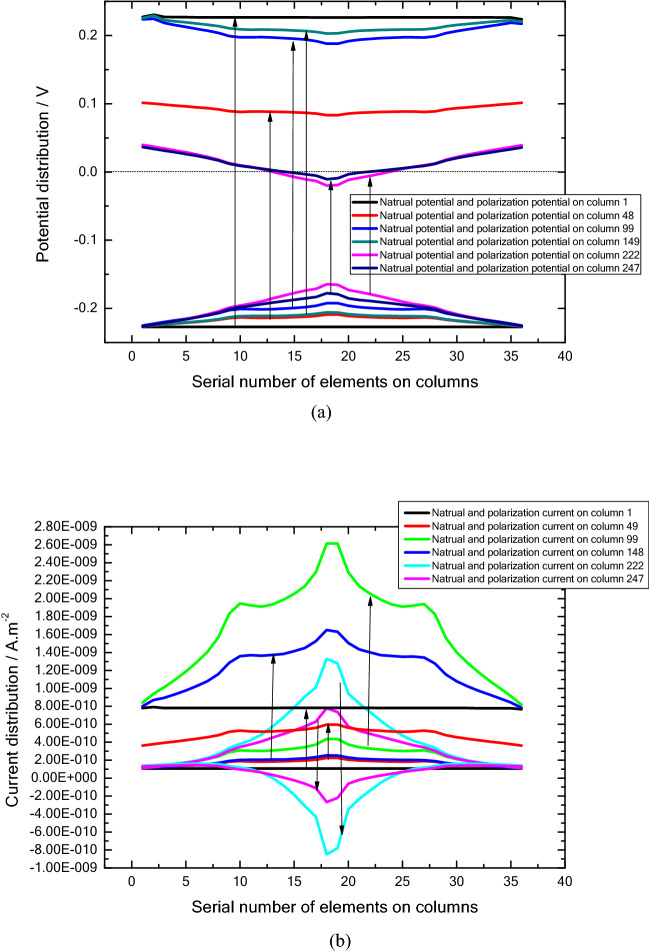
The detailed description of polarization mechanism: (**a**) analysis of polarization of potential, (**b**) analysis of polarization of current.

For columns 222 and 247, cathodic polarization occurs in the middle part. Because the natural corrosion potential at these location are very high (Figs. 7a, 8b, and 11a). Therefore, the element current is mostly in the outflow state, and cathodic polarization is dominant.

The polarization of the elements in these columns has something in common, i.e. the polarization degree near the circumferential edges is higher than that at the middle part. This is because the concentration of oxygen is lower at the edge and the corresponding corrosion potential is also lower.

Driven by the polarization potential, the polarization current is generated, and its distribution is illustrated in Figs. 10b and 11b. In general, anodic polarization causes anodic current while cathodic polarization causes cathodic current and corrosion. The corrosion current is the algebraic sum of natural corrosion and polarization current, as illustrated in Figs. 8c and 10c. It can be seen that the polarization current is basically in the same order of magnitude as the natural corrosion current. Thus, the polarization current presents a significant influence on the final corrosion current distribution. It indicates that the concentration corrosion cannot be ignored in the corrosion analysis. Due to the concentration corrosion, at the location with high original corrosion current, the corrosion current tends to decrease, whereas the corrosion current can increase at the location with low original corrosion current. Additionally, if the solution resistance is not considered, the potentials of all elements will eventually tend to be uniform.

## Conclusions

A two-dimensional DCC model was developed to predict the distribution of corrosion potential and current in the pipeline. The calculation results present a significant influence of concentration corrosion on the overall corrosion of seawater pipeline. The existence of concentration corrosion helps polarize the corrosion potential and subsequently causes the polarization current. The high natural corrosion potential area can cause cathodic polarization and cathodic current, leading to an increase in the corrosion rate. In contrast, the original low natural corrosion potential area can cause anodic polarization and anodic current so that the corrosion rate decreases. The corrosion potential tends to be homogenized due to the differential concentration corrosion. All these findings help clarify the corrosion mechanism in the seawater pipeline with the existence of the differential concentration.

## Methods

### Geometric modeling

Taking pipeline fluid as the research object. The geometry of the fluid is shown in Fig. 2b. The geometry is generated in software COMSOL and exported in format *.x_b.

### Structured grid generation

In order to esTablelish the DCC model successfully, the fluid must be divided into structured grids. This is done in ICEM,a model of Ansys software.The geometry of the fluid(in format *.x_b) was imported into ICEM and structured grid was generated using mapping method. At the same time, the boundary conditions are defined in this model. After meshing, it is saved into MSH format and imported into FLUENT.

### Calculation and data export

In FLUENT, select the type of calculating model (multiphase flow of mixture, turbulence model κ–ε), select the fluid material (the primary phase is water, the secondary phase is oxygen), and then set various boundary conditionss (inlet velocity, oxygen concentration, etc.), and then calculate. After the calculation, UDF technology is used to output the element center coordinates and oxygen concentration close to the wall surface to the file for matlab processing.

### Data processing and display in MATLAB

Use MATLAB script program to read the above data. Then calculate in the following order:

1. According to the formulas (5)–(10) and (24)–(32) , nature corrosion potential, nature corrosion current, polarization resistance and solution resistance of each element are calculated;
2. For Eqs. (18)–(23), the coefficient matrix and constant term matrix are calculated;
3. The polarization corrosion potential was obtained by solving the equations;
4. On this basis, the polarization potential and polarization current are calculated and displayed in graphic form .
